# Development of a Novel Ultrasound-Guided Needle Cricothyroidotomy Device

**DOI:** 10.3390/jcm14165871

**Published:** 2025-08-20

**Authors:** Hidenobu Watanabe, Harumasa Nakazawa, Joho Tokumine, Miki Nagase, Koichiro Saito, Tomoko Yorozu, Kiyoshi Moriyama

**Affiliations:** 1 Department of Anesthesiology, Kyorin University School of Medicine, Mitaka 181-8611, Japan; hidewatanabe@ks.kyorin-u.ac.jp (H.W.); jtokumine@ks.kyorin-u.ac.jp (J.T.); tyorozu@ks.kyorin-u.ac.jp (T.Y.); mokiyo@ks.kyorin-u.ac.jp (K.M.); 2 Department of Anatomy, Kyorin University School of Medicine, Mitaka 181-8611, Japan; mnagase@ks.kyorin-u.ac.jp; 3 Department of Otolaryngology-Head and Neck Surgery, Kyorin University School of Medicine, Mitaka 181-8611, Japan; k-saitoh@ks.kyorin-u.ac.jp

**Keywords:** cricothyroidotomy, ultrasound-guided, CICO, airway management, Seldinger technique

## Abstract

**Background:** Ultrasound-guided identification of the cricothyroid membrane is more accurate than traditional palpation techniques. Additionally, real-time ultrasound-guided puncture is more precise than ultrasound alone. However, no dedicated device currently exists for ultrasound-guided needle cricothyroidotomy. In this study, we aimed to develop and evaluate a novel ultrasound-guided cricothyroidotomy device. **Methods:** A randomized, prospective, crossover simulation study was conducted using a porcine larynx model. Sixteen anesthesiologists and six anesthesia residents participated after receiving video-based and hands-on training. Each participant performed cricothyroidotomy using three methods: ultrasound-guided needle cricothyroidotomy using the novel device (US-G), needle cricothyroidotomy using a commercial cricothyroidotomy kit (QuickTrach^®^), and scalpel incisional cricothyroidotomy after conventional palpation identifying the cricothyroid membrane (Pal-SI). The primary outcome was the puncture success rate. Secondary outcomes included procedure time and tracheal wall injury rates. **Results:** Cricothyroidotomy of the porcine larynx had a success rate of 100% for US-G and Pal-C and 95% for Pal-SI. The US-G procedure time was significantly longer (median: 80 s) than for Pal-C (22 s) or Pal-SI (51 s). No significant differences in the tracheal wall injury rates were noted across methods, and no severe injuries were reported in the US-G group. **Conclusions:** US-G demonstrated a high success rate and good safety profile. Although the procedure time was longer than other methods, its precision may still be beneficial in cases involving anticipated difficult airways. Further clinical validation is warranted.

## 1. Introduction

According to the United Kingdom’s National Audit Project 4, cricothyroidotomies performed by anesthesiologists have a reported success rate of approximately 40%, indicating a relatively low success rate [1]. However, the value of point-of-care ultrasound in airway management has gained increasing recognition [2], with studies showing that ultrasound is more accurate than palpation for identifying the cricothyroid membrane [2,3].

In a previous study, we demonstrated the effectiveness of real-time ultrasound-guided cricothyroid membrane puncture using a simulator mimicking anatomical abnormalities in the patient’s neck [4]. However, that study assessed puncture using only a thin needle under ultrasound guidance and did not assess whether cannulation with a diameter sufficient for ventilation was feasible.

We developed a novel ultrasound-guided cricothyroidotomy kit because commercially available kits are poorly suited for real-time ultrasound use. Visualizing a thick needle like the QuickTrach^®^ (Teleflex Medical, Wayne, PA, USA) under ultrasound is challenging due to skin compression and air trapping. The Melker Emergency Cricothyrotomy Catheter Set^®^ (Cook Medical, Bloomington, IN, USA) has a thin needle but requires a skin incision, limiting its ultrasound compatibility. Other design considerations in the development of ultrasound-guided cricothyroidotomy needles include needle length and safe tubing insertion for ventilation. Excessively long needles risk posterior laryngeal or tracheal wall injury [5]. To address these challenges, we developed a device with adjustable needle length ultrasound visibility and a Seldinger-based system for safe ventilation. In this study, we evaluated the feasibility of this device using an ex vivo porcine larynx model as a simulator for the normal human larynx.

## 2. Materials and Methods

### 2.1. Participants

This study was conducted in accordance with the Declaration of Helsinki and its subsequent amendments and was approved by the local ethics committee (Faculty of Medicine Research Ethics Committee; H29 Kai-7 approval no. 1044-02 and R05-078 approval no. 2209).

The randomized, prospective, crossover simulation study was conducted between October 2023 and June 2024. Participants were anesthesiologists or anesthesia residents. Individuals with prior cricothyroidotomy experience or who declined to participate were excluded. Written informed consent was obtained from all participants.

### 2.2. Sample Size and Power Analysis

To the best of our knowledge, there have been no studies on ultrasound-guided cricothyroidotomy to date. Therefore, assuming a clinically significant difference, we calculated α = 0.05 and estimated the sample size needed to obtain 80% power to be 22 participants.

### 2.3. Development of an Ultrasound-Guided Cricothyroidotomy Kit

**Figure 1:**
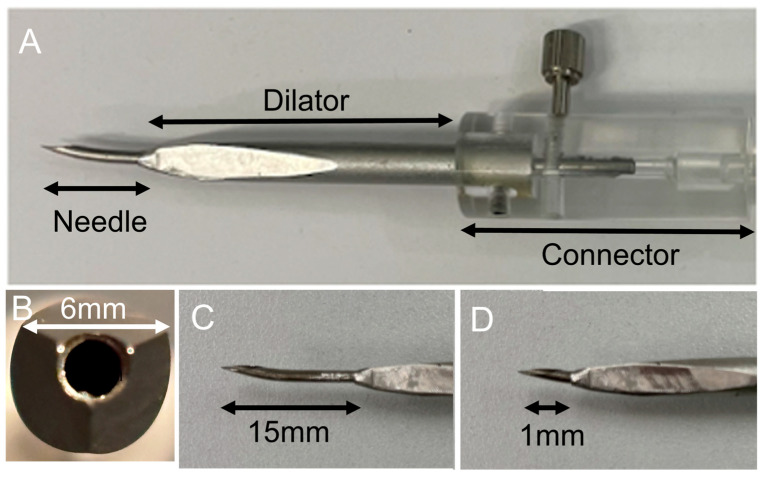
Ultrasound-guided cricothyroidotomy kit. (**A**): Image showing the developed ultrasound-guided cricothyroidotomy kit. The kit includes a 16-G Tuohy needle, dilator, and connector. (**B**): Dilator features a 6 mm diameter structure with a triangular pyramid tip and three cutting edges. The connector links the needle to the dilator and the screw connector adjusts and secures the length of the needle exiting the dilator. (**C**): Effective puncture length of the needle was fixed at 15 mm. (**D**): When the screw connector was completely loosened, the needle was shortened to 1 mm from the tip. However, the needle did not enter the dilator even when the screw connector was entirely loosened.

The device was designed for performing ultrasound-guided puncture of the cricothyroid membrane using a thin puncture needle using the Seldinger technique (Figure 1). First, the needle selected for the ultrasound-guided puncture was a 16 G Tuohy needle with the largest thickness visible on ultrasound. In our previous study using porcine ex vivo larynges, the QuickTrach^®^ (Teleflex Medical, Wayne, PA, USA) with a 25 mm puncture length needle resulted in a 33% posterior wall injury rate. However, a 20 mm puncture length needle, which was 5 mm shorter, led to a 5% posterior wall injury rate [5]. The distance from the skin to the cricothyroid membrane was approximately 1 cm [6]; therefore, the effective puncture length of the developed needle was determined to be 15 mm. A thick cannula was inserted to facilitate ventilation, and the device was designed to allow the enlargement of the puncture site with a dilator after guidewire insertion using the Seldinger method, thereby enabling cannula insertion. The Tuohy needle and dilator were designed as pre-assembled structures to facilitate rapid dilation after puncture. The diameter of the dilator was set at 6 mm based on the average short diameter of the cricothyroid membrane [7]. As the cricothyroid artery, a branch of the superior thyroid artery, crossed the upper edge of the cricothyroid membrane [7], the dilator was shaped as an inverted triangle to avoid damaging the cricothyroid artery, and the dilator was pre-assembled and attached to the puncture needle using a screw connector.

**Figure 2:**
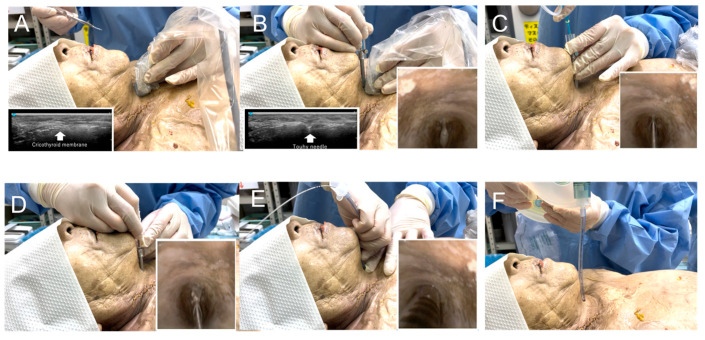
Ultrasound-guided cricothyroidotomy on a cadaver. Images show the cricothyroidotomy performed on a cadaver using an ultrasound-guided cricothyroidotomy kit. Cadavers were fixed in N-vinyl-2-pyrrolidone. Fixation was characterized by the softness and suppleness of the tissues. (**A**): First, the cricothyroid membrane was identified using ultrasound. (**B**): The needle was inserted into the trachea through the cricothyroid membrane under ultrasound guidance, and the bronchoscopy image shows the needle in the trachea. (**C**): Intratracheal needle placement was confirmed by aspirating air using a syringe, after which a guidewire was inserted into the trachea through the needle. (**D**): The cricothyroid membrane was then dilated using the dilator. (**E**): After dilation, the needle and dilator were removed, leaving only the guidewire, and the tracheal tube was inserted into the trachea. (**F**): Ventilation is confirmed by raising the chest wall while squeezing the bag valve mask.

**Figure 3:**
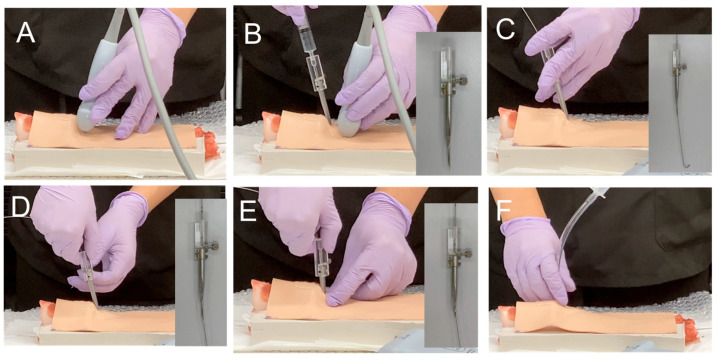
Procedure of the ultrasound-guided cricothyroidotomy. Pictures show the cricothyroidotomy procedure performed on the porcine larynx using an ultrasound-guided cricothyroidotomy kit. (**A**): First, the cricothyroid membrane was identified by ultrasound. (**B**): Under ultrasound guidance, the cricothyroid membrane was punctured with a Tuohy needle. Air aspiration using a syringe confirmed that the needle had entered the tracheal lumen. (**C**): After removing the syringe, the guidewire was inserted into the trachea through the needle. (**D**): The needle and dilator were disconnected by loosening a screw connector. (**E**): The dilator was inserted into the trachea to dilate the skin and cricothyroid membranes. (**F**): An uncuffed tracheal tube (inner diameter, 6 mm) was inserted over the guidewire.

Figure 2 shows a preliminary study of needle cricothyroidotomy in a cadaver [8] using the developed ultrasound-guided cricothyroidotomy kit. A smooth cricothyroid puncture was performed (Video S1). Figure 3 shows the annotated photographs of the device used on a cadaver or porcine larynx.

### 2.4. Study Preparation: Video Lecture and Hands-On Training for Cricothyroidotomy

All participants received instructional videos explaining the three cricothyroidotomy techniques: the novel ultrasound-guided needle cricothyroidotomy device (self-made movie; Supplemental Materials), the commercial needle cricothyroidotomy kit (QuickTrach^®^) [9], and the scalpel incision method [10].

On the day of the experiment, participants reviewed the same instructional videos and were guided through each technique’s steps by the examiner’s demonstration. They then practiced each technique using a porcine larynx, performing each method at least three times to ensure uniformity in mastering the techniques.

### 2.5. Preparation of Porcine Ex Vivo Larynges

The preparation of porcine larynges was performed as previously described [5]. Briefly, larynges were obtained from a commercial meat supplier. For each experiment, larynges were procured in advance and utilized within 2–3 days of delivery. During transportation, the larynges were maintained under chilled conditions and subsequently stored at 3–5 °C until use.

To ensure consistency and stability during the puncture procedures, each larynx was positioned in a custom-designed groove box shaped to accommodate the laryngeal anatomy, as detailed in our previous report [5]. Due to inherent anatomical variation, only larynges that fit securely within the box were included in the study. Larynges that were too small or too large to be firmly stabilized were excluded.

### 2.6. Ultrasound-Guided Cricothyroidotomy

This study compared three cricothyroidotomy techniques (Table 1): ultrasound-guided needle cricothyroidotomy using the novel device (US-G), conventional identification of the cricothyroid membrane using the palpation technique with a commercial needle cricothyroidotomy kit: QuickTrach^®^ (Pal-C), and scalpel incision for cricothyroidotomy after identifying the cricothyroid membrane using the palpation technique (Pal-SI). The participants performed the randomly assigned techniques in sequence using a crossover design. The order of the techniques was determined using a random function in Microsoft Excel. The puncture success rate, procedure time, and tracheal wall injury rate were evaluated using the three techniques. Success was defined as the insertion of the tube into the trachea, whereas failure was defined as either the tube being inserted outside the trachea or exceeding 3 min from the start of the procedure. There was no restriction on the number of puncture attempts participants could perform, provided these were completed within the 3 min time limit. The procedure time was measured from the start of the procedure until the completion of tracheal tube insertion. The examiner assessed tracheal wall injury using a bronchoscope placed inside the porcine larynx to check for any damage. After the procedure, the larynx was dissected and the posterior (or side) wall was inspected with indigo carmine dye to verify any injuries. If damage was found, the severity of the injury was classified on the basis of whether the tracheal wall damage was limited to the mucosa or extended into the muscular layer. The tracheal injury rate was evaluated by tissue observation. The primary outcome was success rate, and the secondary outcomes included procedure time and tracheal wall injury rate.

### 2.7. Statistical Analysis

Numerical values are presented as ratios (%) or as the mean ± standard deviation for normal distributions and as the median (interquartile range) for non-normal distributions. Success and tracheal wall injury rates were assessed using Fisher’s exact test. The procedure time was evaluated using one-way ANOVA, and Sidak’s test was used for post hoc comparisons. The presence or absence of carryover effects was examined using a linear mixed-effects model. Statistical significance was defined as *p* < 0.05. All data were analyzed using GraphPad Prism (ver. 10.4.2, Dotmatics, Boston, MA, USA) and EZR software (ver. 1.68, Jichi Medical University, Saitama, Japan) [11].

## 3. Results

**Figure 4:**
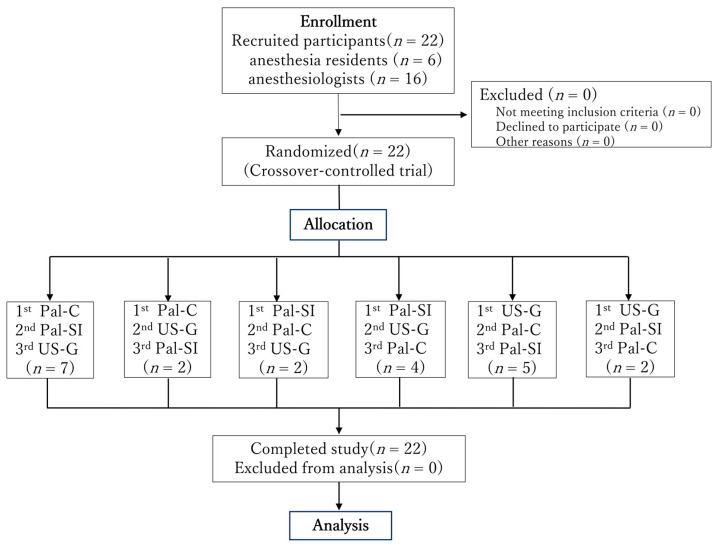
Flow diagram of the study. US-G: cricothyroidotomy using the novel ultrasound-guided needle cricothyroidotomy device, Pal-C: cricothyroidotomy using a commercial cricothyroidotomy kit (QuickTrach^®^) after identifying the cricothyroid membrane using the palpation technique, Pal-SI: scalpel incisional cricothyroidotomy after identifying the cricothyroid membrane using the palpation technique.

**Figure 5:**
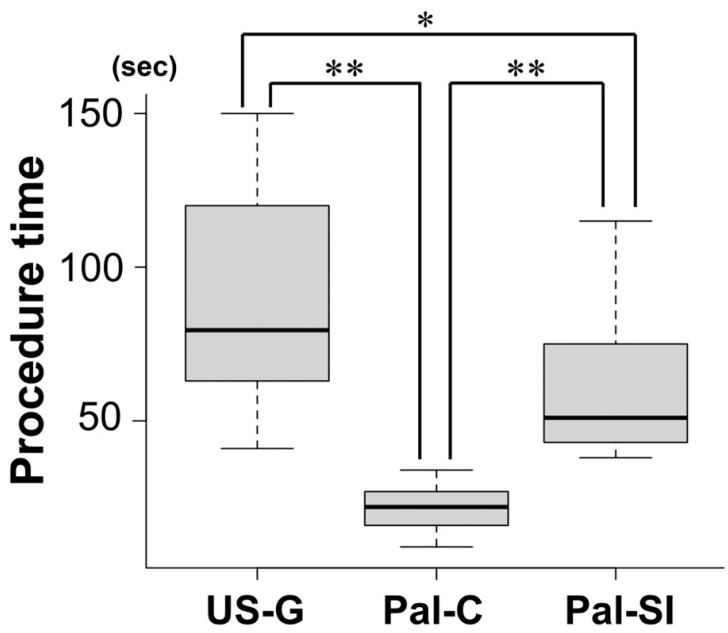
Comparison of procedure times among the three methods. Median (1st quartile, 3rd quartile): Pal-C (22 (16, 27)), Pal-SI (51 (44, 74)), and US-G (80 (63, 115)). Procedure time was significantly shorter for Pal-C compared with both Pal-SI and US-G, and Pal-SI was significantly shorter than US-G. * *p* = 0.02, ** *p* < 0.001.

Sixteen anesthesiologists and six anesthesia residents participated in the study (Figure 4). The success rate was 100% for both the ultrasound guidance and conventional palpation methods and 95% for the scalpel incision method (one failure occurred). However, there were no statistically significant differences among the three methods (Table 2). The median procedure time was 22 s for Pal-C, 51 s for Pal-SI, and 80 s for US-G. A statistically significant difference in procedure time was observed among the three methods (Figure 5). Pal-C had a significantly shorter procedure time than both Pal-SI and US-G (*p* < 0.001), and Pal-SI was significantly shorter than US-G (*p* = 0.02). Tracheal wall injury rates were 18%, 31%, and 23% for the US-G, Pal-C, and Pal-SI groups, respectively. Regarding the damage severity evaluated using the tissue dying method, severe damage reaching the tracheal posterior wall muscle layer was observed: ultrasound-guided, 0%; Pal-C, 5%; and Pal-SI, 14%. However, no statistically significant differences were observed between the methods. No carryover effect was observed on the success rate and procedure time (Table S1).

### Subgroup Analysis

There were no statistically significant differences in success rates, tracheal wall injury rates, or procedure times between anesthesiology residents and anesthesiologists (Table S2).

## 4. Discussion

We developed a novel ultrasound-guided cricothyroidotomy device and evaluated its feasibility and safety using a porcine larynx model, which simulates the normal structure of a human larynx. The success rate of the novel ultrasound-guided cricothyroidotomy device was comparable to that of conventional palpation-based technique using a commercial needle cricothyroidotomy kit: QuickTrach^®^, and the scalpel incision method, which is currently considered the standard in the Difficult Airway Society (DAS) guidelines [12]. The time required for the novel ultrasound-guided cricothyroidotomy device was 30–60 s longer than that required for traditional techniques, likely due to the additional time needed for the ultrasound-based identification of the cricothyroid membrane and the multiple steps required by the Seldinger technique. In a “cannot intubate cannot oxygenate” situation in which every minute counts, a surgical airway must be secured with both certainty and speed. Previous studies have compared cricothyroidotomy performed by anesthesiologists using the Melker Emergency Cricothyrotomy Catheter Set^®^ (Cook Medical, Bloomington, IN, USA) (skin incision, needle puncture, and cannula insertion using the Seldinger technique) using an animal laryngeal model similar to that used in this study, in which the scalpel incisional cricothyroidotomy took a median of 45–54 s, and the Melker Emergency Cricothyrotomy Catheter Set^®^ took a median of 90–100 s [13,14]. In contrast, our ultrasound-guided needle cricothyroidotomy device had a median procedure time of 80 s, suggesting it may be faster than the Melker Emergency Cricothyrotomy Catheter Set^®^.

In an obese animal model, the median time for scalpel incisional cricothyroidotomy was reported to be 89–109 s [15,16]. These data indicated that even rapid techniques may take nearly 100 s to perform in the presence of anatomical abnormalities such as obesity (without laryngeal deviation or rotation). These results suggest that the surgical incisional technique may not be completed quickly in a clinical setting if anatomical abnormalities impede airway security. Additionally, if the trachea deviates, the cricothyroid membrane may not lie beneath the incision site. To prevent this, identification of the cricothyroid membrane using ultrasonography may be necessary. In such cases, the time required for the surgical incision technique is extended.

Difficult airways can be classified into two types: unanticipated and anticipated. A predicted difficult airway is typically associated with anatomical abnormalities of the neck, which complicates the identification of the cricothyroid membrane using conventional palpation techniques. We previously demonstrated that real-time ultrasound guidance for needle cricothyroidotomy may be more effective than simple identification alone when using ultrasound neck simulators with anatomical abnormalities [4]. In cases of anticipated difficulty in securing airways with anatomic abnormalities, a “double setup” [17] is recommended, in which awake intubation and emergency cricothyroidotomy are prepared simultaneously. In such cases, the cricothyroid membrane can be identified in advance using ultrasound. Ultrasound identification of the cricothyroid membrane typically takes approximately 20 s, regardless of the body mass index [18,19]. One major drawback of the ultrasound-guided needle cricothyrotomy device developed in this study is the number of steps involved in the Seldinger technique. The problem with the equipment is that the needle and dilator are separated by turning a screw connector. If other methods could be improved to enable quick detachment, this would save time. These time-saving improvements will be the subject of future studies.

**Figure 6:**
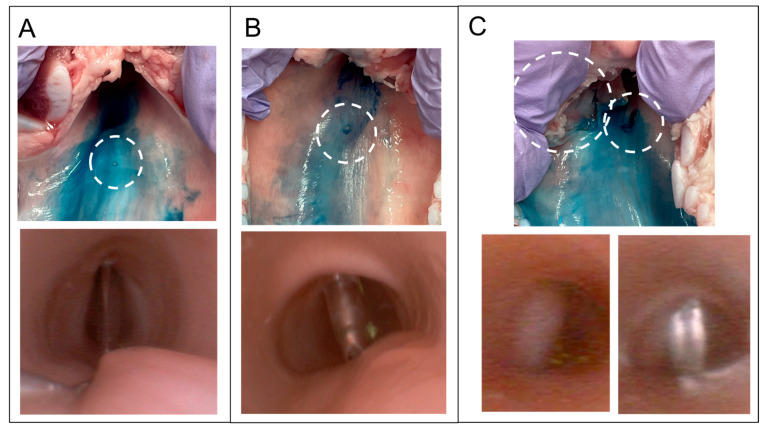
Tracheal wall injury. In each technique, the upper photographs show a dissected porcine larynx examined for posterior or lateral tracheal wall injuries. The porcine larynx was incised along the sagittal line at the center of the anterior surface and 0.4% indigo carmine solution was sprayed onto the internal tracheal surface. White dotted circles indicate the sites of injury. The lower photographs show injury to the larynx, as observed by a bronchoscope set in the larynx during the experiment. (**A**): Cricothyroidotomy using a novel ultrasound-guided needle cricothyroidotomy device. Although indentations caused by the needle tip were detected, no lacerations were observed. (**B**): Cricothyroidotomy using a commercial cricothyroidotomy kit (QuickTrach^®^) after identifying the cricothyroid membrane using the palpation technique. A pinpoint laceration of the mucosa caused by the needle was detected. (**C**): Scalpel incisional cricothyroidotomy after identification of the cricothyroid membrane by palpation. The images show two deep lacerations in the muscular layer in one case. The lower left picture shows a bougie migrating under the lateral tracheal wall, which causes a lateral tracheal laceration (left dotted circle in the upper image). The lower right picture shows the stabbing of the posterior tracheal wall by a scalpel, which causes laceration of the posterior tracheal wall (right dotted circle in the upper image).

Severe tracheal injuries involving the muscle layer were observed using both the needle cricothyroidotomy kit (QuickTrach^®^) and the scalpel incisional cricothyroidotomy. In contrast, ultrasound-guided needle cricothyroidotomy caused only minor mucosal injuries and no significant damage to the trachea, although the difference was not statistically significant (Figure 6). These minor mucosal injuries likely resulted from mucosal stretching (“tenting”) during dilation, which caused the Tuohy needle to press against the posterior wall [5]. When the needle is inserted vertically, the cricoid cartilage located behind the cricothyroid membrane may help prevent deeper injury, limiting damage to the mucosal puncture. In comparison, the QuickTrach^®^ device is operated at 45° as the tip perforated the cricothyroid membrane and then advanced until it was inhibited by the stopper. This sharp tip touches the posterior tracheal wall, which lacks protection from the cricoid cartilage’s posterior plate, and causes injury extending into the muscle layer [5].

In scalpel incisional cricothyroidotomy, injuries to the muscle layer are caused either by the bougie migrating into the submucosa of the lateral tracheal wall or by a deeper-than-expected scalpel incision (Figure 6). Injury to the muscle layer should be avoided due to the risk of tracheal perforation [20]. One possible reason for bougie migration is insufficient cutting by the scalpel blade [21]. Even if the scalpel is advanced to a sufficient depth, mucosal tenting may occur if it does not completely cut the mucosa [5]. In such cases, only the tip of the scalpel entered the tracheal lumen. Consequently, the bougie may slide along the mucosal surface instead of penetrating it. Tracheal injury is reported to be seven times more likely after a failed first bougie insertion attempt than after a successful attempt [13], possibly because the bougie is redirected into gaps created by prior mucosal disruption. There are also reports of other complications such as tracheal injury or even parts of the scalpel blade left inside the body during cricothyroidotomy [22]. Thus, even this seemingly safe scalpel technique carries the risk of unexpected complications. However, since this study was conducted by anesthesiologists unfamiliar with scalpel incisions, the results are not directly applicable to cricothyrotomy, as performed by surgeons. Furthermore, cricothyrotomy performed by surgeons differs from the method recommended by the DAS guidelines [12]. This is because, after incising the cricothyroid membrane, the surgeon opens the window with forceps or a hook, checks the lumen of the trachea, and inserts a tracheal tube.

This study had some limitations. First, the conventional cricothyroidotomy needle was not examined under ultrasound guidance. Second, the study did not explore the potential to reduce complications through enhanced incision training. Third, the study did not assess difficult airway simulators. It should also be noted that ultrasound-guided puncture may be more challenging in patients with difficult airways, and the performance of the novel device in such scenarios was not evaluated in this study. Additionally, the 3 min time limit for each procedure may have affected the results, as allowing more time could potentially change the outcomes. Finally, the relatively small sample size may limit the generalizability of the findings.

In future studies, it will be necessary to assess the effectiveness of this novel device in patients with predicted difficult airways, such as those with morbid obesity or anatomical abnormalities of the neck, to further evaluate its clinical applicability.

## 5. Conclusions

The developed ultrasound-guided needle cricothyrotomy device has a high success rate and safety profile. The only drawback, however, is the longer procedure time compared to other methods. However, this drawback may not be problematic in cases of difficult airway security.

## Figures and Tables

**Table 1:** Comparison of characteristics among the three cricothyroidotomy approaches.

	US-G	Pal-C	PAL-SI
Accuracy of CTM identification	○ US guidance	△ Palpation	△ Palpation
Size of the incision	○ Small	○ Small	△ Moderate
Reliability of airway access	○ High	△ Low	◎ High
Technical complexity	× Multi-step	○ Simple	△ moderate
Risk of posterior wall injury	○ Low	△ High	○ Low
Risk of bleeding	○ Low	○ Low	△ High
Key characteristics	Accurate CTM identification Less invasive Multiple cumbersome steps	Simple procedure Small incision High risk for wall injury	High reliability of airway access High invasive Risk for wall injury and bleeding

This table summarizes the key characteristics and parameters of three cricothyroidotomy approaches: ultrasound-guided needle cricothyroidotomy using the novel device (US-G), conventional identification of cricothyroid membrane using palpation technique with a commercial needle cricothyroidotomy kit: QuickTrach^®^ (Pal-C), and scalpel incision for cricothyroidotomy after identifying the cricothyroid membrane using palpation technique (Pal-SI). ◎ = Most preferred feature, ○ = Preferred feature, △ = Less-preferred feature, × = Unfavorable feature.

**Table 2:** Success rate, procedure time, tracheal wall injury rate, and severe wall injury rate.

Measurements	US-G	Pal-C	Pal-SI	*p* Value
Success rate % (*n*)	100 (22)	100 (22)	95 (21)	1.0
Procedure time m (1st, 3rd) (s)	80 (63, 115)	22 (16, 27)	51 (44, 74)	<0.001
Tracheal wall injury rate % (*n*)	18 (4)	31 (7)	23 (5)	0.68
Severe wall injury rate % (*n*)	0 (0)	5 (1)	14 (3)	0.31

m: median, 1st: 1st quartile, 3rd: 3rd quartile, US-G: cricothyroidotomy using the novel ultrasound-guided needle cricothyroidotomy device, Pal-C: cricothyroidotomy using a commercial cricothyroidotomy kit (QuickTrach^®^) after identifying the cricothyroid membrane using the palpation technique, Pal-SI: scalpel incisional cricothyroidotomy after identifying the cricothyroid membrane using the palpation technique.

## Data Availability

The datasets generated in this study are available from the corresponding author upon request.

## Supplementary Materials

The following supporting information can be downloaded at: https://www.mdpi.com/article/10.3390/jcm14165871/s1, Video S1: Ultrasound-guided cricothyroidotomy on a cadaver. Table S1: Effects of Technique, Sequence, and Session on Procedure Time: Linear Mixed Model Analysis. Table S2: Subgroup Analysis by Experience Level: Comparison of Procedure Outcomes Between Anesthesiologists and Anesthesia Residents.
