# Supplementary material for: Development of a Novel Ultrasound-Guided Needle Cricothyroidotomy Device
Source: J Clin Med. 2025 Aug 20;14(16):5871. doi: 10.3390/jcm14165871 (PMC12387471; doi:10.3390/jcm14165871)
Supplement: Supplementary file 1 [file jcm-14-05871-s001.zip › jcm-3765088-supplementary done.pdf]

Table S1. Effects of Technique, Sequence, and Session on Procedure Time: Linear Mixed Model Analysis.

Fixed effect		Estimate	Std. Error	*p* Value
Technique (Reference: US-G)	Pal-C	−63.56	7.48	<0.001
	Pal-SI	−20.61	7.46	0.009
Sequence (Reference: US-G→Pal-C→Pal-SI)	US-G→Pal-SI→Pal-C	−27.58	19.23	0.171
	Pal-C→US-G→Pal-SI	−32.58	19.23	0.110
	Pal-C→Pal-SI→US-G	−16.20	13.92	0.261
	Pal-SI→US-G→Pal-C	−4.12	14.89	0.786
	Pal-SI→Pal-C→US-G	−6.08	19.23	0.756
Period (Reference: 1st session)	2nd session	−1.86	3.74	0.622

In this study, a linear mixed-effects model based on a crossover design was used to evaluate differences in procedure time among three techniques (Pal-C, Pal-SI, US-G). The model included technique, sequence, and period as fixed effects, and participants as random effects. Neither sequence (order of implementation) nor period (first or second session) had a statistically significant effect ($p > 0.1$). US-G: cricothyroidotomy using the novel ultrasound-guided needle cricothyroidotomy device, Pal-C: cricothyroidotomy using a commercial cricothyroidotomy kit (QuickTrach®) after identifying the cricothyroid membrane using the palpation technique, Pal-SI: scalpel incisional cricothyroidotomy after identifying the cricothyroid membrane using the palpation technique

Table S2. Subgroup Analysis by Experience Level: Comparison of Procedure Outcomes Between Anesthesiologists and Anesthesia Residents.

		**Anesthesiologists (*n* = 16)**	**Anesthesia Residents (*n* = 6)**	***p* Value**
US-G	Success rate% (*n*)	100 (16)	100 (6)	-
	Tracheal wall injury rate% (*n*)	25 (4)	0 (0)	0.54
	Procedure time, m (1st, 3rd) (sec)	79 (61,120)	80 (67, 91)	0.25
Pal-C	Success rate% (*n*)	100 (16)	100 (6)	-
	Tracheal wall injury rate% (*n*)	25 (4)	50 (3)	0.33
	Procedure time, m (1st, 3rd) (sec)	22 (17, 26)	20 (15, 28)	0.33
Pal-SI	Success rate% (*n*)	93.8 (15)	100 (6)	1.0
	Tracheal wall injury rate% (*n*)	12.5 (2)	50 (3)	0.10
	Procedure time, m (1st, 3rd) (sec)	49 (41, 60)	74 (57,105)	0.16

This table presents the results of a subgroup analysis comparing anesthesiologists and residents for each cricothyroidotomy technique. Procedure time was analyzed using one-way ANOVA for each technique, and success rate and tracheal wall injury rate were analyzed using Fisher's exact test. No statically significant difference was observed in procedure time, success rate, or tracheal wall injury rate between anesthesiologist and residents within any individual technique.
